# Characterization of the Physiological Response following* In Vivo* Administration of* Astragalus membranaceus*

**DOI:** 10.1155/2016/6861078

**Published:** 2016-04-12

**Authors:** Karen Denzler, Jessica Moore, Heather Harrington, Kira Morrill, Trung Huynh, Bertram Jacobs, Robert Waters, Jeffrey Langland

**Affiliations:** ^1^Southwest College of Naturopathic Medicine, Tempe, AZ 85282, USA; ^2^Arizona State University, Biodesign Institute, Tempe, AZ 85287, USA

## Abstract

The botanical,* Astragalus membranaceus*, is a therapeutic in traditional Chinese medicine. Limited literature exists on the overall* in vivo* effects of* A. membranaceus* on the human body. This study evaluates the physiological responses to* A. membranaceus* by measuring leukocyte, platelet, and cytokine responses as well as body temperature and blood pressure in healthy individuals after the* in vivo* administration of* A. membranaceus*. A dose-dependent increase in monocytes, neutrophils, and lymphocytes was measured 8–12 hours after administration and an increase in the number of circulating platelets was seen as early as 4 hours. A dynamic change in the levels of circulating cytokines was observed, especially in interferon-*γ* and tumor necrosis factor-*α*, IL-13, IL-6, and soluble IL-2R. Subjective symptoms reported by participants were similar to those typically experienced in viral type immune responses and included fatigue, malaise, and headache. Systolic and diastolic blood pressure were reduced within 4 hours after administration, while body temperature mildly increased within 8 hours after administration. In general, all responses returned to baseline values by 24 hours. Collectively, these results support the role of* A. membranaceus* in priming for a potential immune response as well as its effect on blood flow and wound healing.

## 1. Introduction

Botanical medicines are plant-derived products which have increasingly come under significant investigation for their potential therapeutic applications [1–3]. The use of botanical medicines worldwide is increasing significantly [4, 5]. A 2007 National Institute of Heath Survey revealed that 44% of Americans 50–59 years of age and 38% of adults less than 50 years of age have used some form of Complementary and Alternative Medicine (CAM), including botanical medicines [6]. Approximately $14.8 billion dollars in out-of-pocket expenditures for nonvitamin, nonmineral, natural products were spent that year (compared to $47.6 billion spent on pharmaceutical drugs) [7]. Due to the growing demand for alternative therapies and the general public notion that botanical medicines are safe, some physicians prefer or are considering referral to CAM specialists for their expertise [8–10]. However, evidence based characterization is typically limited regarding many of these therapies, justifying the need for further research.

Originally described in Shen Nong's Classic of Materia Medica over two thousand years ago, the botanical* Astragalus membranaceus* (AM) has been used extensively in traditional Chinese medicine to support and enhance the immune system, to treat various conditions, including viral infection, fatigue, decreased appetite, debility, nonhealing wounds, liver and kidney disease, and cancers [11, 12]. Traditionally, AM is made into a decoction in which pieces of root were boiled into soups and then removed prior to consumption.

The presumptive active constituents of AM include polysaccharides, saponins, flavonoids, and astragalosides [13, 14]. Recent evidence has also suggested an active component role of lipopolysaccharides provided by endosymbiotic bacteria present on the root of AM [15, 16].* Astragalus* polysaccharides (APS) have demonstrated immunopotentiating properties such as increased murine B-cell proliferation and cytokine production [17]. Numerous* in vitro* studies and limited* in vivo* studies and clinical trials have demonstrated intriguing indications for the use of AM, particularly as an immunomodulator to prevent and treat heart disease, nephritis, bacterial infection, and viral illnesses (especially respiratory infections and chronic hepatitis) and as an adjunct therapy for cancer, HIV, and atopic disease [15, 18–24]. Several animal studies have shown the ability of AM to restore and enhance immunologic function in the cases of either immunosuppression or infection including HSV, HIV, HBV, and viral myocarditis [16, 22–27]. The antiviral and wound healing properties of AM are proposed to be indirect via modulation of proinflammatory cytokines inducing leukocyte and platelet mobilization. Current research in animal models suggests that AM may have a significant clinical effect on cell proliferation and wound healing [28–30]. Although significant research has been conducted on AM,* in vivo* studies are limited. The research presented provides an evaluation of the physiological response to AM following* in vivo* administration of this botanical.

## 2. Materials and Methods

### 2.1. Botanical Extract Preparation

Dried* Astragalus membranaceus* root slices were purchased from Mayway Corporation (Oakland, CA). Dried AM was validated using herbal pharmacopoeia monographs. Six hundred grams of dried AM was ground in a 1 gallon stainless steel Hamilton Beach blender, transferred to a clean amber colored gallon glass jar, and 2220 milliliters of boiling distilled water was added to the ground root. After six hours, 780 mL of 190 proof ethanol was added for a final ratio of 1 : 5 (weight of botanical to volume of liquid). The mixture was kept at room temperature for 3 weeks, followed by separation of the liquid portion from the solid herb portion using a mechanical press. The extracted liquid was filtered using unbleached paper filters, pooled, and dispensed in amber colored bottles. To eliminate any physiological responses due to ethanol, the original 25% ethanol based AM extract was vacuum-dried for 3 hours. Final ethanol concentrations were measured to be 2–4%. A vehicle control sample was prepared from 25% ethanol that was similarly dried for 3 hours. For standardization purposes, a sample of the extract was dried and found to have a concentration of nonvolatile solutes of 92.6 mg/mL extract. Since definitively active constituents present in AM are unknown, we cannot calculate the concentration of active constituent(s) present in the extract. Therefore, this value serves as a reference measure for relative activity.

### 2.2. Participants

This case series study included 2 healthy males (29 yo and 47 yo) and 2 healthy females (24 yo and 27 yo). Criteria for healthy individuals included the absence of known chronic disease, the absence of illness including HIV and HCV, and no use of any medications at the time of the study. Participants were informed that they must be without symptoms of illness at the time of the study and that they must adhere to a controlled diet for 4 days prior to beginning the study (including no alcohol or use of known immunomodulatory foods). The study was approved and overseen by the Arizona State University and Southwest College of Naturopathic Medicine (SCNM) Institutional Review Boards (Protocol 208-11). Each participant received and completed a written informed consent form prior to participation in the study. Most data was acquired from the 47 yo male and 24 yo and 27 yo females; however a 29 yo male replaced the 27 yo female during toxicity testing due to relocation.

### 2.3. *In Vivo* Administration

The ethanol-reduced AM extract or vehicle was administered to three healthy subjects at indicated doses and relative to body weight. Doses included 0.25 mL/kg, 0.75 mL/kg, and 1.5 mL/kg. The AM extract was administered sublingually over a period of 20 minutes followed by ingestion. Trials occurred on separate days with at least 4 weeks in between trials.

Rationale for dosages used is as follows: for acute conditions, doses of AM often range from 1 to 25 g/day. For this study, we wanted to investigate acute changes over a 24-hour period related to the immune response. Therefore, based on average 70 kg adult, the highest dose of 1.5 mL AM extract/kg body weight was calculated with an average adult receiving an extract from 20 g dried AM. Similarly, the 0.75 mL/kg and 0.25 mL/kg doses were based on an average adult receiving extract from 10 g to 3.3 g dried AM, respectively.

### 2.4. Venipuncture

Blood was collected into heparinized tubes (BD vacutainer cell preparation tube with sodium heparin) 0, 4, 8, 12, and 24 hours after administration of the AM extract. Samples were sent to an external lab (Lab Corps) for routine processing including flow cytometry and cytokine assay. Arizona State University and the Southwest College of Naturopathic Medicine (SCNM) Institutional Review Boards approved the collection and processing of all blood samples.

### 2.5. Blood Analysis

Blood sent to external labs was processed through standard cell counting and flow cytometry, and a full blood profile was done which included the following: white blood cell populations, including total white blood cells, lymphocytes, monocytes, neutrophils, and platelets, and lymphocyte subpopulations including total T-cells (CD3+), T-helper cells (CD3+CD4+), T-cytotoxic cells (CD3+CD8+), B-cells (CD19+), and NK-cells (CD56+). A cytokine assay was also performed by the third-party lab via ELISA and included the following cytokines: IL-1*β*, IL-2, sIL-2R, IFN-*γ*, IL-4, IL-5, IL-6, IL-8, IL-10, IL-13, IL-12, and TNF-*α*. Liver (AST, ALT, bilirubin, and ALP) and kidney (potassium, sodium, BUN, creatinine, and BUN/creatinine ratio) panels were tested to assess potential toxicity at 1, 12, and 24 h after ingestion of AM or vehicle.

### 2.6. Physiological Responses

The subjective physiological reactions experienced by each participant were reported at each blood draw. Symptoms were reported and then rated subjectively on an intensity scale of 1–10 (10 highest intensities). Blood pressure and body temperature (taken orally) were recorded by laboratory technicians at each blood draw. Physiologic responses were completed with both the AM trials and the vehicle control trial.

### 2.7. Statistical Analysis

Statistical analyses were performed using SPSS*™* Statistical Analysis Software. Differences between time points (0 versus 12 hours and 0 versus 24 hours) were analyzed using paired 2-tailed “*t*” tests and were considered statistically significant if *p* < 0.05.

### 2.8. Ethics Statement

All research involving human participants was approved by the Southwest College of Naturopathic Medicine Institutional Review Board (Protocol: 208-11). Informed consent was obtained from all participants and all clinical investigations were conducted according to the principles expressed in the Declaration of Helsinki.

## 3. Results

The majority of leukocytes in the body are located in organs of the lymphatic system or connective tissues proper. Circulating white blood cells represent a fraction of the total white blood cell population in the body, and changes from baseline values are useful in interpreting activity of the immune system. For example, inflammation can result from infection or various disease states and cause known characteristic changes in circulating leukocytes. To define any specific changes induced by the* in vivo* administration of AM, an analysis of leukocyte populations including total white blood cells, peripheral blood mononuclear cells (lymphocytes and monocytes), and polymorphonuclear cells (neutrophils) was completed. The observed effects were measured over a 24-hour time period, after* in vivo* administration of AM. This analysis was conducted on 3 separate occasions, each for varying doses of AM including 0.25 mL/kg, 0.75 mL/kg, and 1.5 mL/kg. A control test was done with 1.5 mL/kg vehicle.

**Figure 1:**
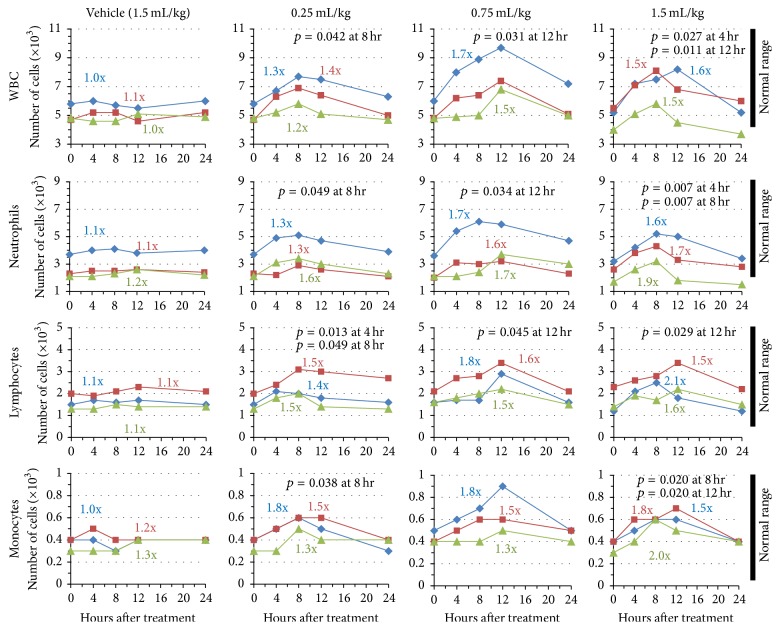
AM effect on white blood cell populations. Subjects 1, 2, or 3 (indicated by blue, red, and green lines, resp.) were administered vehicle or varying concentrations of AM extract (0.25, 0.75, and 1.5 mL extract/kg body weight). Blood draws were performed 0, 4, 8, 12, and 24 hours after administration to measure total cell population numbers. Cells measured include total white blood cells, neutrophils, lymphocytes, and monocytes. Peak fold changes relative to baseline (*t* = 0 hours) are indicated. Normal physiological ranges of the different cell populations are indicated by black bars.

As Figure 1 indicates, treatment with the vehicle control had almost no effect on absolute numbers of leukocytes while treatment with AM demonstrated statistically significant increases in cell numbers above initial/baseline values; *p* values were typically significant at 8 and 12 hours. Along with increases in cell numbers, atypical lymphocytes were notably present 8 and 12 hours after treatment (classified by increased granularization, increased size, and chromatin decondensation) and indicate lymphocyte activation. The observed increases in cell number also appeared to be dose-dependent and this was consistent across all subjects (compare 0.25 mL/kg dose with 0.75 mL/kg dose). Fold changes were calculated to best represent the magnitude of change that occurred over the 24-hour period and Figure 1 shows the maximal fold change for each individual subject. Total WBCs increased on average (based on *n* = 3) 1.3x above baseline values at the lowest dose and 1.53x at the highest dose (*p* values 0.042 and 0.011, resp.). Similarly, in comparing 0.25 mL/kg, 0.75 mL/kg, and 1.5 mL/kg doses, neutrophils increased from an average (based on *n* = 3) of 1.4x, 1.68x, and 1.73x above baseline values; lymphocytes increased by 1.46x, 1.63x, and 1.73x, and monocytes increased by 1.53x, 1.53x, and 1.76x above baseline values, respectively. Furthermore, as doses increased, the data also typically became more significant. For example increases in numbers of neutrophils were associated with *p* values, at each of the three doses of AM, of 0.049, 0.034, and 0.007, respectively.

The timing of these changes is noteworthy and consistent. In all subjects, maximal peak values were obtained between 8 and 12 hours. In all cases, baseline values returned by 24 hours after administration of AM. Although increases in cell population were observed following AM administration, cell numbers never exceeded ranges regarded as normal for the human population (normal range indicated in the figure). It is clear that while changes to leukocyte populations were significant compared to baseline values, neither leucopenia nor leukocytosis were observed.

**Figure 2:**
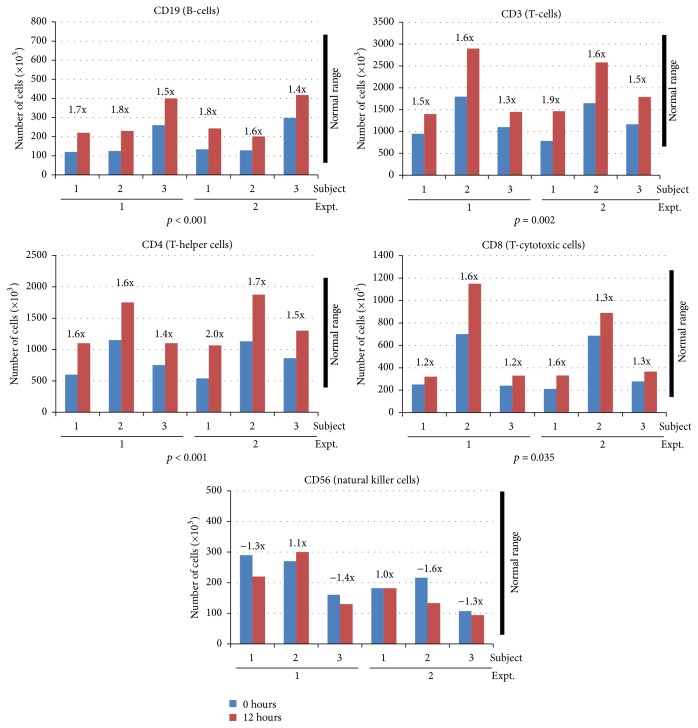
AM effect on lymphocyte subset populations. Subjects (1, 2, or 3) were administered AM at a 1.5 mL extract/kg body weight dose. Blood draws were performed 0 (blue bars) and 12 (red bars) hours after administration and lymphocyte subset cell populations were measured. The experiment was repeated twice. Peak fold changes relative to baseline (*t* = 0 hours) are indicated. Normal physiological ranges of the different cell populations are indicated by black bars.

We next sought to characterize changes in numbers of lymphocyte population subsets circulating in the blood. For this experiment, subjects were administered the highest dose of AM (1.5 mL/kg) on two separate occasions and lymphocyte populations were measured 0 and 12 hours after administration. As shown in Figure 2, increases in T- and B-cells including total CD3+, CD3+CD4+, CD3+CD8+, and CD19+ lymphocytes were observed. CD19+ (B-cells) increased on average 1.66x and 1.60x above baseline values for trials 1 and 2, respectively (*p* < 0.001). Similarly, the average increases for trials one and two demonstrated that total CD3+ cells increased by 1.46x and 1.66x (*p* = 0.002), CD3+CD4+ (T-helper cells) increased by 1.53x and 1.73x (*p* < 0.001), and CD3+CD8+ (T-cytotoxic cells) increased by 1.33x and 1.5x (*p* = 0.035). Collectively, fold changes ranged from 1.2x to 2.0x above baseline values in these cell populations. While increases are statistically significant from baseline values (*p* values including both trials range from *p* < 0.001 to 0.035 between the subsets), all cell population values remained within normal physiological limits. This trend of increases in circulating B- and T-cell subsets suggests that AM may be mobilizing these cells. Conversely, natural killer (NK) cells (CD56+) were shown to mildly decrease or remain unchanged. Subjects responded very similarly between the two separate experiments shown in Figure 2 adding confidence to the physiological effect of AM. For example, compare the initial (*t* = 0 hrs) and final (*t* = 12 hrs) levels of CD3+ cells for subject 2 between trials 1 and 2.

**Figure 3:**
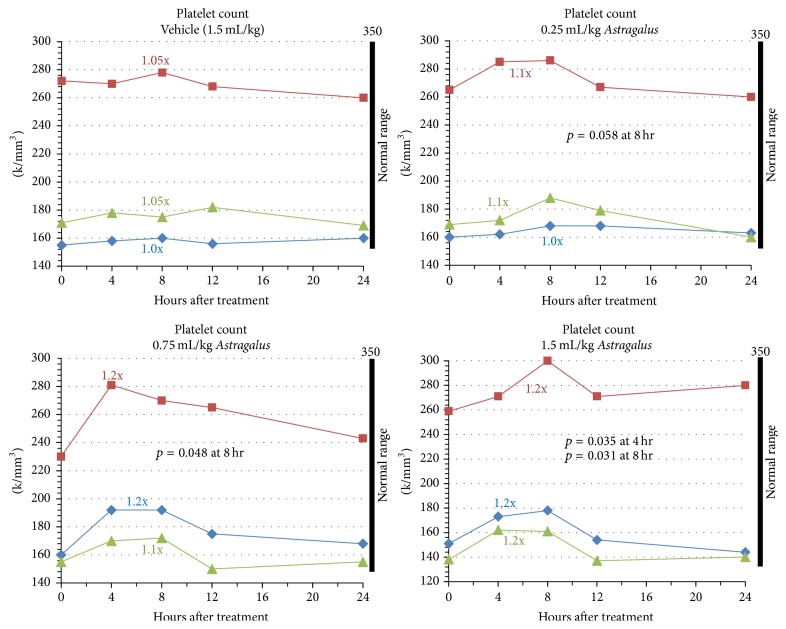
AM effect on circulating platelets. Subjects 1, 2, or 3 (indicated by blue, red, and green lines, resp.) were administered vehicle or varying concentrations of AM extract (0.25, 0.75, and 1.5 mL extract/kg body weight). Blood draws were performed 0, 4, 8, 12, and 24 hours after administration to measure total platelet cell numbers. Peak fold changes relative to baseline (*t* = 0 hours) are indicated. Normal physiological ranges of the different cell populations are indicated by black bars.

Since AM is clinically reported for use in damaged tissues such as diabetic wound healing, we next evaluated changes to platelet counts in relation to increasing doses of AM* in vivo* [31, 32]. Wound healing is a complex and dynamic process involving a highly regulated sequence of biochemical and cellular events. Platelets contribute to this process by providing the initial hemostasis that occurs after tissue injury and by producing growth factors responsible for the regeneration of damaged tissues. As seen in Figure 3, platelet counts were moderately increased by statistically significant values in the 0.75 mL/kg dose and 1.5 mL/kg dose. In addition, a dose-dependent trend reflected in absolute increases from baseline values and significance was observed. Average fold increases in circulating platelets for the three increasing doses were 1.07x (*p* = 0.058 at 8 h), 1.17x (*p* = 0.048 at 8 h), and 1.20x (*p* = 0.035 at 4 h; *p* = 0.031 at 8 h) above baseline values, respectively. While the two highest doses resulted in significant increases, changes occurred within normal physiological ranges and there was no evidence of thrombocytopenia or thrombosis at any point in time. The analysis also demonstrated that peak values are obtained between 4 and 8 hours after administration of AM. Comparatively, peak values for leukocyte counts were observed between 8 and 12 hours. A return to baseline values was observed across all subjects by 12 to 24 hours after administration of AM.

**Figure 4:**
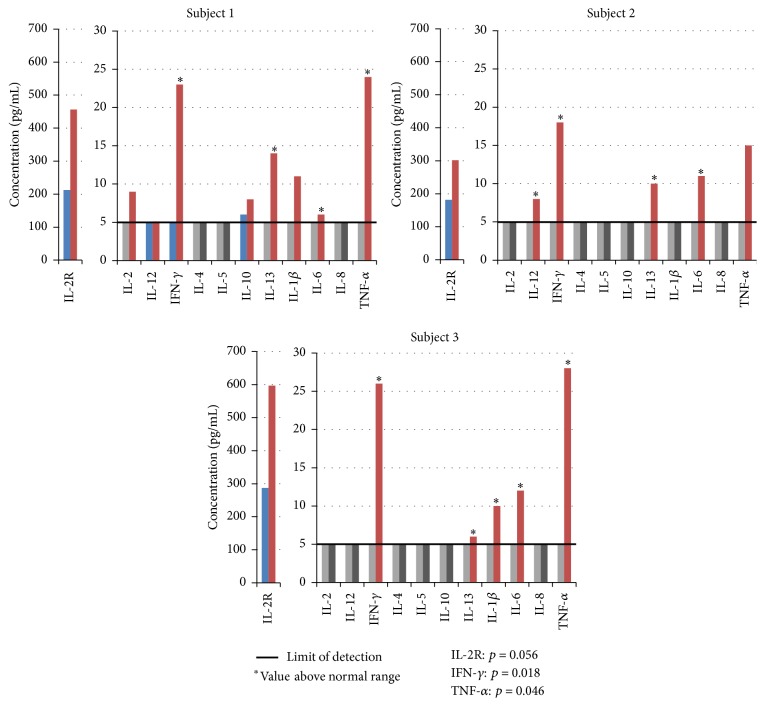
AM effect on cytokine response. Subjects (1, 2, or 3) were administered AM at 1.5 mL extract/kg body weight dose. Blood draws were performed 0 (blue or light grey bars) and 12 (red or black bars) hours after administration and specific cytokine levels measured. Detection limits for all cytokines were 5 pg/mL (5 pg/mL limit is indicated by the black line). Blue and red bars indicate values at or above 5 pg/mL. Light grey and black bars indicate values less than 5 pg/mL (nondetectable). Cytokine levels above normal physiological ranges are indicated by *∗*. *p* values for IL-2R, IFN-*γ*, and TNF-*α* are shown.

Since* in vivo* administration of AM has been reported to differentially modulate cytokine activity, we continued our analysis by reviewing intercellular communication between immune populations through changes to circulating cytokines [33]. As Figure 4 illustrates, this analysis was done after the administration of the highest dose of AM at 1.5 mL/kg and assay of serum for various cytokines including interleukins (IL-2, IL-4, IL-5, IL-6, IL-8, IL-10, IL-12, and IL-13), soluble IL-2 receptor (IL-2R), interferon gamma (IFN-*γ*), and tumor necrosis factor alpha (TNF-*α*) at 0 and 12 hours. Circulating levels of these cytokines were quantified with a minimum limit of detection at 5 pg/mL. Increases above the limit of detection were seen in IL-2R, IFN-*γ*, IL-6, IL-13, and TNF-*α* across all three subjects. Induction of IL-1*β* was observed in two subjects, while induction of IL-2, IL-10, and IL-12 was observed in only one subject each. IL-4 and IL-5 were not induced above the limit of detection in any participant. The most significant changes were seen in IL-2R, IFN-*γ*, and TNF-*α* (*p* = 0.056, *p* = 0.018, and *p* = 0.046, resp.). These results are consistent with the promotion of a Th1 immune response by AM due to the increase of IFN-*γ* and TNF-*α* and the absence of IL-4.

The potential for AM to cause liver or kidney toxicity following ingestion was assessed to determine safety at the highest dose (1.5 mL/kg). For all liver enzyme and kidney function tests performed, no statistically significant differences were measured at 12 and 24 h after administration (data not shown). Similarly, the vehicle had no effect on liver and kidney tests (data not shown).

**Figure 5:**
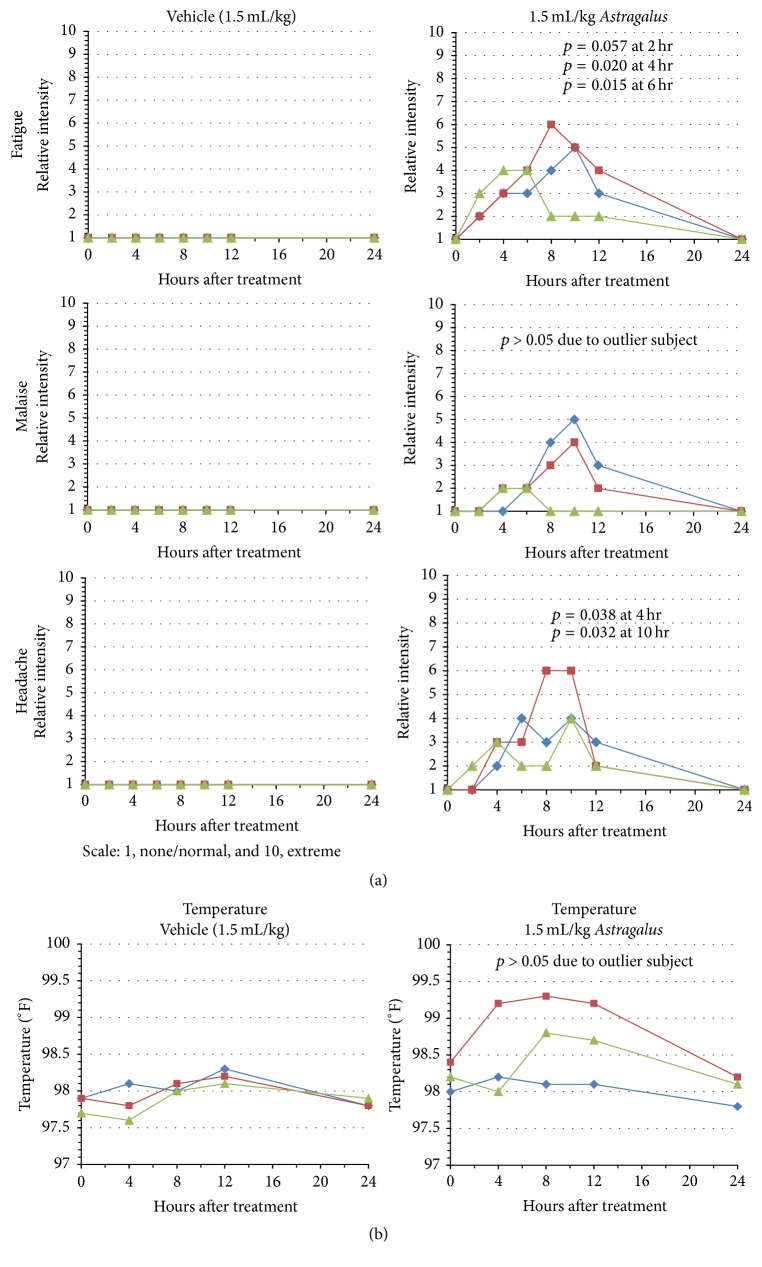
Physiological responses in subjects following administration of AM. Subjects 1, 2, or 3 (indicated by blue, red, and green lines, resp.) were administered vehicle or 1.5 mL extract/kg body weight dose of AM extract. (a) Symptoms of fatigue, malaise, and headache were recorded at 0, 2, 4, 6, 8, 10, 12, and 24 after administration. Values were subjective ranging from 1 to 10 (1, none/normal, and 10, extreme/severe). (b) Body temperature was measured orally 0, 4, 8, 12, and 24 hours after administration of the extract.

In addition to these studies, we evaluated symptomatic outcomes following administration of AM. Physiological symptomatic responses were dose-dependent with the most dramatic results observed at the highest AM dose (data not shown). As shown in Figure 5(a), typical “flu-like” symptoms were reported by all participants. The symptoms included fatigue, malaise, headache, and a reduced capacity to mentally focus. Symptoms were first reported between 2 and 4 hours and reached maximal values between 6 and 10 hours after ingestion of AM. All participants demonstrated a decline in symptoms by 12 hours and by 24 hours no symptoms were reported by any individual. The average peak intensity ratings for these three symptoms were 3.67/10 (malaise), 4.67/10 (headache), and 5/10 (fatigue); although peak intensities occurred at different time points. Statistical significance, as represented by *p* values, for headache and fatigue ranged from 0.015 to 0.057. While ratings for malaise showed trending in two out of three subjects, one subject experienced only very minor fatigue and for a much shorter duration than the others. Thus the *p* value was >0.05. These symptoms are consistent with those typically reported in proinflammatory sickness [34, 35]. No other significant physiological symptoms were reported by the subjects.

Our study also monitored changes to body temperature over the 24-hour time period after administration of the 1.5 mL/kg dose of AM. Compared to the vehicle control, two of the three subjects showed significant trending in increased body temperatures (Figure 5(b)). The other subject did not demonstrate a change in body temperature. The maximum value noted was 99.3°F and may be interpreted as a mild fever response. Maximum temperatures were observed by 8 hours after administration of AM and temperatures returned to baseline values by 24 hours.

**Figure 6:**
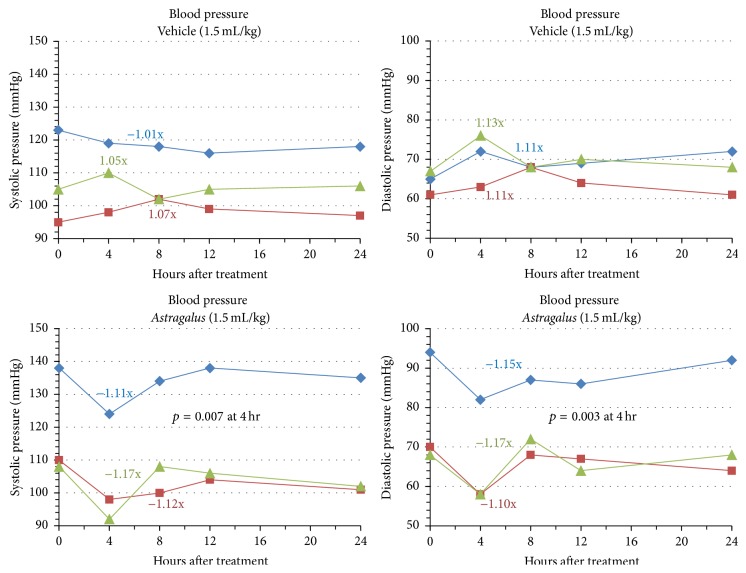
AM effects on blood pressure. Subjects 1, 2, or 3 (indicated by blue, red, and green lines, resp.) were administered vehicle or 1.5 mL extract/kg body weight dose of AM extract. Blood pressure (systolic and diastolic) was measured 0, 4, 8, 12, and 24 hours after administration of the extract.

To further understand the effects of AM on blood flow, we also measured blood pressure at each blood draw during the 1.5 mL/kg trial. As shown in Figure 6, a decrease in systolic pressure (ranging from −1.11x to −1.17x baseline values) by an average of −1.13x below baseline values was seen consistently across participants 4 hours after administration. In contrast, the vehicle control led to an average increase of +0.37x above baseline values for systolic blood pressure. Similarly, participants also showed consistent decreases in diastolic blood pressure ranging from −1.10x to −1.17x with an average of −1.14x in response to AM 4 hours after ingestion. Again these results differ from those seen with the vehicle control with increased diastolic pressure of 1.16x baseline values on average. Systolic and diastolic changes were significant with *p* values of 0.007 and 0.003 at 4 hours, respectively. With regard to the timing of response, participants followed very similar trends where maximal changes were observed at the 4-hour time point. This result was similar to the time response observed with changes to circulating platelets. A return to baseline values was seen in all participants 8–24 hours after ingestion of AM. Despite significant decreases in systolic and diastolic values, the lowest values seen did not drop below normal physiological limits of systolic pressure of 90 mm Hg or diastolic pressure of 60 mm Hg and hypotension was not induced at any point in time.

## 4. Discussion

Our data supports a correlation between the purported physiological effects of the immunomodulatory botanical, AM, and changes in cytokine gene expression and immune cell circulation.* In vivo*, AM induced significant elevations in both total and specific leukocyte populations but changes remained within normal physiological limits. Specifically, CD3+, CD3+CD4+ and CD3+CD8+, and CD19+ lymphocyte populations were induced while natural killer (CD56+) cell populations were unchanged. In addition, total neutrophil and monocyte numbers increased suggesting a global mobilization of leukocytes into the blood stream following AM.

Previous data from microarray analysis after the* in vitro* treatment of peripheral blood mononuclear cells (PBMCs) with AM suggested that proinflammatory and Th1 specific cytokines were primarily upregulated [15]. In this regard, AM has been reported to modulate cytokine expression from Th2 toward Th1 in cancer and chronic viral infection [36, 37]. Our present* in vivo* data demonstrate a potential for priming toward a Th1 response where AM lead to the significant induction of Th1 cytokines, IFN-*γ* and TNF-*α*, along with a moderate induction of proinflammatory IL-6. The presence of IL-6 mediates fever and induces the acute phase response in the liver as well as acting as a differentiation factor to B-cells, monocytes, and macrophages and can act to regulate the development of a Th1 immune response through the induction of SOCS-1 and IL-4 [38]. However, our data do not show induction of IL-4 in serum following AM administration even though IL-6 is induced. IFN-*γ* leads to increased Th1 responses during infection or immunization and inhibits Th2 cell proliferation. IFN-*γ* is involved in macrophage activation, the generation of cell-mediated immunity, and upregulation of antiviral and antimicrobial effector molecules, and, in conjunction with TNF-*α*, can induce NF-*κ*B responsive genes synergistically [39]. TNF-*α* itself is a pyrogen and an acute phase protein that promotes the inflammatory response, especially during diapedesis, and is highly expressed in Th1-activated T-cells. Alternately, with induction of the Th2 cytokine, IL-13 was observed. IL-13 plays a nonredundant role with IL-4 in generating resistance to gastrointestinal parasites and intracellular organisms, increases mucus production, and generates IgE responses [40]. Despite being traditionally considered a Th2 cytokine, recent characterization of IL-13 has shown that it can be produced by IFN-*γ*+ Th1 cells and by Th17 cells [41]. Finally, soluble IL-2R was significantly induced in all subjects. It is normally expressed on antigen activated T lymphocytes and leads to T-cell proliferation. Its secretion indicates the presence of activated T-cells and can be an indicator of infection, neoplasms, and autoimmunity [42]. The levels of soluble IL-2R were increased 1.5–2.5-fold following AM treatment; however levels are lower than those seen in patients with chronic hepatitis infection (1.6–4-fold), acute EBV infections (7-fold), or B-cell cancer patients (up to 6.4-fold) but are similar to levels seen in autoimmune patients with rheumatoid arthritis (2-fold) [43–46].

Both direct and immune modulatory activities may explain the reported antibacterial effects of AM. It is recognized that when IFN-*γ* and TNF-*α* bind to their respective macrophage receptors, they stimulate the release of nitric oxide which results in increased destruction of bacteria [47]. In addition to cytokines, polysaccharides isolated from AM are also potent inducers of nitric oxide [48]. Alternately, polysaccharides isolated from AM have recently been shown to suppress Treg function by downregulating the production of IL-10 in a murine model of bacterial sepsis [49]. This may explain, at least in part, some of the clinical success for AM reported in the literature regarding antibacterial activity.

Our analysis of immune stimulation by AM related to white blood cell populations was of interest. As the dose of AM increased, absolute numbers of total leukocytes, neutrophils, lymphocytes, and monocytes also increased. Concurrently, increasing doses resulted in higher statistical significance as seen by an associated decrease in *p* values. Therefore, immune stimulation by AM is likely producing a dose-dependent response to changes in white blood cell populations. It is noteworthy that these changes remain within normal physiological ranges since therapeutic applications would not necessarily desire pathological immune responses such as leukocytosis or leukopenia. Historically, AM has been used to support and enhance the immune system, for instance in conditions of debility and viral infection. Given the rapid peak in circulating WBC populations we suspect that AM stimulates a mobilization of the immune cells into circulation from peripheral tissues/organs rather than hematopoietic formation of new cells from the bone marrow. It is possible that AM acts to stimulate immune cell mobilization leading to broad, systemic scanning of potential antigens as if “positioning” the immune cells for activity. It is then reasonable to infer that following mobilization, with no antigen present to continue activation of the immune cascade, the response is subsequently diminished and circulating cell populations are normalized by 24 hours after treatment. Surprisingly, administration of AM did not increase circulating levels of NK-cells. NK-cells are an important component in the antiviral immune response and are typically activated in response to IL-2, IL-12, IL-15, IL-18, and CCL5. Of these cytokines, we only assayed IL-2 and IL-12, each of which was induced in separate subjects. These results may suggest some level of specificity to the innate response induced by AM.

Regarding blood flow and wound healing, previous literature and anecdotal evidence suggests that AM may be associated with an increased risk of bleeding tendencies, improved endothelial cell function, improved blood glucose control, improved cardiovascular function overall, and blood pressure lowering effects [50–52]. Our results demonstrated a minor increase in platelets within 4–8 hours after administration of AM; a faster response than that observed in the leukocyte population. Like the leukocyte populations, there was an increase in absolute numbers of circulating platelets with higher dosing of AM. Our previous* in vitro* AM microarray studies identified putative genes induced by AM which are associated with angiogenesis, wound healing, and blood pressure modulation. It is known that the role of platelets is to provide the initial hemostasis that occurs after tissue injury and to produce growth factors responsible for the regeneration of damaged tissues such as platelet derived growth factor (PDGF), transforming growth factor (TGF), and endothelial growth factor (EGF). Our cytokine analysis showed significant increases in IL-6, which is involved in PDGF-induced cell proliferation [53]. Similarly IL-13 is known to induce the release of TGF from epithelial cells [54]. While our studies support the notion that AM may induce wound healing through changes in specific cytokine populations and numbers of circulating platelets, further studies are needed to determine a more complete understanding of the relationships between cytokine induction, cellular responses, and clinical results.

Our results demonstrated a decrease in blood pressure following administration of AM. Clinically, both whole botanical applications and isolated constituents of AM, such as astragaloside IV, have been shown to be beneficial in ischemic heart disease, heart failure, myocardial infarction, and relief of anginal pain [55]. This may be due in part to a vasodilatory effect leading to the observed reduction in both systolic and diastolic pressure. Indeed, IL-6, IL-13, and TNF-*α* are also known inducers of vasodilation [56–58].

An increase in body temperature has been historically recognized since ancient times as an indication of infection or inflammation. Current research demonstrates that T-cytotoxic cells are affected by mild hyperthermia through changes to cell membranes, which then mediate cell function and differentiation [59]. There is little to no significant data in the literature on the effect of AM on body temperature. Since individuals deviate only slightly from the standard value of 98.6°F, our results suggest a mild effect on body temperature due to AM. Two out of three of our subjects demonstrated minor although significant increases in temperature to a maximum value of 99.3°F in one subject. Furthermore, there is a clear trend in those subjects which mirrors that of the WBC response with maximum values between 8 and 12 hours and a subsequent return to baseline by 24 hours. Increases in activated white blood cells leads to the production of endogenous pyrogens such as IL-1, IL-6, and TNF-*α*. Our results likely indicate an increase in temperature as part of the coordinated steps of normal immune activation.

To understand this response even further, subjective symptoms including fatigue, malaise, and headache were observed to occur soon after ingestion of AM and returned to baseline values by 24 hours. Subjects also reported a lack of mental focus. The early presence of these symptoms (often before 4 hours) indicates a likely prodrome type of response which parallels the notion that immune cell populations are being induced for wide spread systemic scanning. TNF-*α* is an acute phase response proinflammatory cytokine involved in pain activation including headache, malaise, and fatigue [60, 61]. Proinflammatory cytokines, IL-6 (all 3 patients) and IL-1*β* (2 patients), were also measurable and, together with TNF-*α*, likely resulted in symptoms associated with immune activation.

Our study provides analyses of the physiological responses following administration of AM to a limited number of subjects. To the best of our knowledge, no other study has elucidated such an overall response under this specific group of parameters and in healthy individuals. Since there has been an increase in the use of botanical therapies, including herbal medicine, health food, and cosmetics, this research is relevant to CAM practitioners, primary care physicians, and other medical doctors alike. The significant potential for therapeutic and clinical application as well as the growing interest for scientific evidence and expertise in the use of botanical medicine creates a clear need for* in vivo* analysis and further characterization of AM.
